# Women’s opinion on the justification of physical spousal violence: A quantitative approach to model the most vulnerable households in Bangladesh

**DOI:** 10.1371/journal.pone.0187884

**Published:** 2017-11-21

**Authors:** Raaj Kishore Biswas, Nusma Rahman, Enamul Kabir, Farabi Raihan

**Affiliations:** 1 School of Agricultural, Computational and Environmental Sciences, University of Southern Queensland, Toowoomba, Queensland, Australia; 2 Department of Statistics, Jagannath University, Dhaka, Bangladesh; 3 Institute of Research and Training (ISRT), University of Dhaka, Dhaka, Bangladesh; Swinburne University of Technology, AUSTRALIA

## Abstract

Bangladesh is a culturally conservative nation with limited freedom for women. A number of studies have evaluated intimate partner violence (IPV) and spousal physical violence in Bangladesh; however, the views of women have been rarely discussed in a quantitative manner. Three nationwide surveys in Bangladesh (2007, 2011, and 2014) were analyzed in this study to characterize the most vulnerable households, where women themselves accepted spousal physical violence as a general norm. 31.3%, 31.9% and 28.7% women in the surveys found justification for physical violence in household in 2007, 2011 and 2014 respectively. The binary logistic model showed wealth index, education of both women and their partner, religion, geographical division, decision making freedom and marital age as significant household contributors for women’s perspective in all the three years. Women in rich households and the highly educated were found to be 40% and 50% less likely to accept domestic physical violence compared to the poorest and illiterate women. Similarly, women who got married before 18 years were 20% more likely accept physical violence in the family as a norm. Apart from these particular groups (richest, highly educated and married after 18 years), other groups had around 30% acceptance rate of household violence. For any successful attempt to reduce spousal physical violence in the traditional patriarchal society of Bangladesh, interventions must target the most vulnerable households and the geographical areas where women experience spousal violence. Although this paper focuses on women’s attitudes, it is important that any intervention scheme should be devised to target both men and women.

## Introduction

The attitude towards women is not homogeneous all over the world; differences are even observed within industrialized countries, where women have more freedom than in third world nations [1–4]. Developed and developing nations have their own unique characteristics but in both of these cultural environments, women are increasingly able to voice their rights and participate in public sectors [5, 6]. The traditional conservative nature of the south-east Asian households is not encouraging for women to express their opinion on most occasions and often they are forced to accept a subordinate role in the society [7–10]. Interestingly, a large percentage of males and females in various countries condone justification of physical spousal violence in specific contexts [11]. There are cases reported where women justified physical spousal violence more than men [12]. Importantly, because Bangladesh is a culturally conservative nation with limited freedom for women [13–15], the assessment of their opinion remains a challenge.

Because Bangladesh is a conservative country, women are generally confined to the house, especially in sub-urban and rural areas [16, 17]. Women’s autonomy is low and empowerment is limited [18]. Bangladeshi women are habituated by their vulnerable socioeconomic position to see their role as an obedient wife, who raises children and does the household chores [19, 20]. The prevailing social dogma compels women to undertake the inferior roles in the family; this hegemony restricts their potential and ultimately leads to devaluation of their own opinion [21]. These limitations foster an attitude of acceptance of IPV among women who may see it as part of their daily lives. This study plans to characterize the most vulnerable households where women accept this traditional ideology.

In order to understand women’s attitudes to physical spousal violence in Bangladesh, cultural context must be considered. The prevalence rate of spousal violence towards married women varies from 32% to 72%, according to the recent studies [22–24]. These rates are not uncommon as approximately 50% of women in low income countries believe that beating wives, or physical spousal violence, is justified [25]. The contemporary patriarchy in Bangladesh condemns women to be a property of their father, later husband and any *disobedience* is considered punishable [26–29]. Moreover, the prevalence of dowry forces women to marry at a young age, as older brides require a higher dowry before marriage. Often times the amount of dowry determines both a woman’s status in the family and the value of her opinion in the in-laws’ house, particularly in rural areas and urban slums [10, 30]. However, a contradiction among women regarding IPV was observed by Sato et al., 2015 [31], who found that women tend to accept IPV in specific contexts, and these contexts contradict their general statement on IPV. Because of these ambiguities, we considered four specific contexts for spousal physical violence and women’s opinion of each.

A number of studies have evaluated intimate partner violence (IPV) and spousal physical violence in Bangladesh [32–34]. However, the views of women were rarely discussed in a quantitative manner. When considering the impact of cultural diversity, there is a need for more context-dependent studies that focus on a woman’s situation as a wife [22, 23, 35, 36]. In particular, why would a woman find any justification in being beaten by her partner/husband? Three nationwide surveys in Bangladesh (2007, 2011, and 2014) were assessed in this study to characterize the most vulnerable households, where women themselves accepted spousal physical violence as a general norm. We found and discussed that household economic insolvency, illiteracy and early marriage are the major reasons behind this perception.

## Methods

### Ethical approval

This article does not contain any studies with human participants performed by any of the authors. The Bangladesh demographic and health Surveys were approved by ICF Macro Institutional Review Board and the National Research Ethics Committee of the Bangladesh Medical Research Council. A written consent about the survey was given by participants before interview. All identification of the respondents was dis-identified before publishing data. The secondary data sets analyzed during the current study are freely available upon request from the DHS website at http://dhsprogram.com/data/available-datasets.com. Searching ‘Bangladesh DHS, 2011’ in the DHS website will provide the survey data set.

### Data description

Bangladesh Demographic and Health Survey (BDHS), a nationally representative cross-sectional survey, has been conducted in Bangladesh since 1993 in collaboration with Demographic and Health Survey (DHS), operated by Measure DHS+ [37, 38]. A list of enumeration areas (EAs) from the census is used as the sampling frame [38]. Two-stage stratified cluster sampling techniques are applied for this survey. In the first stage, 600 EAs (or clusters) were selected using a proportional to size (PPS) sampling method. In the second stage, an equal probability systematic sampling method is applied to draw an average of 30 households from each cluster. We used the three most recent BDHS of 2007, 2011 and 2014, where only the females were considered as respondents and the temporary (de jure) residents were excluded in the sample. The total sample size for the surveys were 9173, 16500, 16620 respectively, after removing the missing vales (< 10%) and temporary (de jure) residents.

The response variable (binary) in this study is the opinion of respondents (women) regarding physical spousal violence by their husband. They were asked if they think it is justified to beat the wife, if she (a) goes out without telling her husband, (b) neglects the children (c) argues with her husband and (d) refuses to have sex with her husband. As the answers were binary (Yes/No), we compiled all the negative responses as ‘No’ and any positive response as ‘Yes’. Hence, if a respondent found justification for spousal physical violence in any circumstances, they were marked as receptive to the idea of spousal physical violence at home, whereas those who said no in all four cases were considered to be strictly opposed to any forms of physical violence. 31.3%, 31.9% and 28.7% of women in the surveys found justification for physical violence in the household in 2007, 2011 and 2014 respectively.

The opinion for the justification of physical spousal violence (binary outcome) was fitted to household socioeconomic status (SES). BDHS provides a long range of variables on public health and household characteristics and some among them were chosen for this study. These variables are the most common SESs used in Bangladesh public health analyses, based on the BDHS (Table 1), particularly for explaining women’s health [39–42]. Household wealth index (poorest, poorer, middle, rich, richest), education of women and partner (none, primary., secondary, higher), religion (Islam, others), residence (rural, urban), division (Barisal, Chittagong, Dhaka, Khulna, Rajshahi, Rangpur, Sylhet), decision making freedom (herself/joint, others), media exposure (none, at least one) and age of marriage (before 18, at 18 or more) are the covariates considered to be fitted with the outcome. The household wealth index was calculated based on asset variables using the principal component analysis (PCA) by DHS.

**Table 1:** Frequency distribution of SES and outcome variable over three surveys.

SES variables	BDHS 2007 (N = 9173)	BDHS 2011 (N = 16500)	BDHS 2014 (N = 16620)
**Wealth Index**			
Poorest	1484 (16.2%)	2792 (16.9%)	2964 (17.8%)
Poor	1669 (18.2%)	3092 (18.7%)	3135 (18.9%)
Middle	1741 (19.1%)	3199 (19.4%)	3391 (20.4%)
Richer	1860 (20.3%)	3510 (21.3%)	3511 (21.1%)
Richest	2419 (26.4%)	3907 (23.7%)	3619 (21.8%)
**Education of respondent (women)**			
No education	2862 (31.2%)	4039 (24.5%)	3646 (21.9%)
Primary	2799 (30.5%)	4953 (30.0%)	4859 (29.1%)
Secondary	2777 (30.3%)	6082 (36.9%)	6459 (38.9%)
Higher	735 (8.0%)	1422 (8.6%)	1656 (10%)
**Education of partner**			
No education	2991 (32.6%)	4618 (28%)	4510 (27.1%)
Primary	2467 (26.9%)	4543 (27.5%)	4594 (27.6%)
Secondary	2381 (26.0%)	4851 (29.4%)	4976 (29.9%)
Higher	1334 (14.5%)	2488 (15.1%)	2540 (15.3%)
**Religion**			
Islam	8251 (89.9%)	14654 (88.8%)	15009 (90.3%)
Others	922 (10.1%)	1846 (11.2%)	1611 (9.7%)
**Residence**			
Rural	5713 (62.3%)	10790 (65.4%)	10938 (65.8%)
Urban	3460 (37.7%)	5710 (34.6%)	5682 (34.2%)
**Division**			
Barisal	1218 (13.3%)	1947 (11.8%)	2001 (12.0%)
Chittagong	1601 (17.5%)	2667 (16.2%)	2671 (16.1%)
Dhaka	1974 (21.5%)	2838 (17.2%)	2875 (17.3%)
Khulna	1438 (15.7%)	2461 (14.9%)	2399(14.4%)
Rajshahi	1747 (19%)	4714 (28.6%)	4745 (28.6%)
Sylhet	1195 (13%)	1873 (11.4%)	1929 (11.6%)
**Decision making freedom**			
Self or joint	4229 (46.1%)	7785 (47.2%)	7565 (45.5%)
Others	4944 (53.9%)	8715 (52.8%)	9055 (54.5%)
**Media exposure**			
None	4285 (46.7%)	5524 (33.5%)	6041 (36.3%)
At least one	4888 (53.3%)	10976 (66.5%)	10579 (63.7%)
**Marital age**			
Before 18 years	7444 (81.2%)	12806 (77.6%)	12615 (75.9%)
At 18 or more	1729 (18.8%)	3694 (22.4%)	4005 (24.1%)
**Outcome variable**			
Beating is justified	2871 (31.3%)	5256 (31.9%)	4770 (28.7%)
Beating not justified	6302 (68.7%)	11244 (68.1%)	11850 (71.3%)

Two variables were defined by the authors. ‘Decision making freedom’ was based on three questions asked during interviews: whether the respondent takes decisions regarding a) her own health care, b) large household purchases, and c) visits to family or relatives; or whether someone else (mostly partner) takes them on her behalf. We formulated a dichotomous variable, where one scale considered the respondent’s participation in the decision (either alone or joint) and the other scale did not involve her. Similarly, ‘Media exposure’ was compiled from respondent’s regular interactions with newspaper, radio and television. If they are exposed to any one (or more), they were scaled as exposed to ‘at least one’ (Table 1).

### Statistical algorithm

A bivariate analysis was conducted to overview the covariates and the outcome variable. A binary regression model was fitted to the outcome variable with the household SESs. This model is one of the most stable models to analyze dichotomous outcome variables, with clear interpretations and simpler general logistics model assumptions [43, 44]. It has numerous applications in public health studies because of high goodness of fit [45, 46]. All statistical analyses were performed in *R* (*version* 3.4.1). Generally, p-value of 0.05 is considered the threshold of significant association. However, according to Benjamin et al., 2017 [47], the threshold of 0.05 should be replaced by 0.005 due to the increasing evidence concerning non-reproducible research claims of significant effects or relationships within the scientific community. Therefore, we will interpret variables as significant only when the p-value is less than 0.005 and also shows consistency with the relevant confidence interval.

## Results

The bivariate relationship between SESs and women’s opinions over the surveys are shown in Table 2. The binary logistic model provided the effect size and confidence intervals of the contributions of each household SES on women’s opinions on physical spousal violence justification. Wealth index, education of both women and their partner, religion, geographical division, decision making freedom and marital age were found as the significant factors in all the three years (Table 2). Interestingly, the residence of the respondents (urban/rural) was not a significant factor for women’s opinions. Respondents’ age (14–49 years) and their partners’ age were homogeneously distributed throughout the data set and did not have any impact on women’s opinion of physical violence in three models.

**Table 2:** Binary logistic model fitted with household SES to women’s opinion regarding physical spousal violence justification.

SES variables	Respondent’s opinion on beating wives by their partner (N = 9173)								
	BDHS 2007			BDHS 2011			BDHS 2014		
	Not justified (%)	Justified (%)	OR (95% C.I.)	Not justified (%)	Justified (%)	OR (95% C.I.)	Not justified (%)	Justified (%)	OR (95% C.I.)
**Wealth Index**									
Poorest	948 (63.9%)	536 (36.1%)	1.00	1647 (59%)	1145 (41%)	1.00	1885 (63.6%)	1079 (36.4%)	1.00
Poor	1063 (63.7%)	606 (36.3%)	0.97 (0.84, 1.13)	1927 (62.3%)	1165 (37.7%)	0.91 (0.81, 1.01)*	2047 (65.3%)	1088 (34.7%)	0.97 (0.87, 1.08)*
Middle	1130 (64.9%)	611 (35.1%)	0.92 (0.79, 1.07)	2101 (65.7%)	1098 (34.3%)	0.85 (0.76, 0.95)*	2399 (70.7%)	992 (29.3%)	0.83 (0.73, 0.93)*
Richer	1264 (68%)	596 (32%)	0.88 (0.75, 1.04)	2449 (69.8%)	1061 (30.2%)	0.76 (0.68, 0.86)*	2539 (72.3%)	972 (27.7%)	0.85 (0.75, 0.97)*
Richest	1897 (78.4%)	522 (21.6%)	0.68 (0.56, 0.82)*	3120 (79.9%)	787 (20.1%)	0.55 (0.47, 0.63)*	2980 (82.3%)	639 (17.7%)	0.60 (0.52, 0.70)
**Education of respondent (women)**									
No education	1893 (66.1%)	969 (33.9%)	1.00	2475 (61.3%)	1564 (38.7%)	1.00	2374 (65.1%)	1272 (34.9%)	1.00
Primary	1840 (65.7%)	959 (34.3%)	1.03 (0.91, 1.16)	3184 (64.2%)	1773 (35.8%)	0.97 (0.88, 1.06)	3263 (67.2%)	1596 (32.8%)	0.93 (0.84, 1.03)
Secondary	1914 (68.9%)	863 (31.1%)	1.09 (0.94, 1.27)	4359 (71.7%)	1723 (28.3%)	0.83 (0.74, 0.94)*	4779 (74.0%)	1680 (26.0%)	0.79 (0.71, 0.89)*
Higher	655 (89.1%)	80 (10.9%)	0.48 (0.35, 0.65)*	1226 (86.2%)	196 (13.8%)	0.49 (0.39, 0.59)*	1434 (86.6%)	222 (13.4%)	0.52 (0.42, 0.64)*
**Education of partner**									
No education	1972 (65.9%)	1019 (34.1%)	1.00	2843 (61.6%)	1775 (38.4%)	1.00	2964 (65.7%)	1546 (34.3%)	1.00
Primary	1593 (64.6%)	874 (35.4%)	1.05 (0.93, 1.19)	2937 (64.6%)	1606 (35.4%)	1.01 (0.92, 1.11)	3115 (67.8%)	1479 (32.2%)	0.99 (0.90, 1.09)
Secondary	1634 (68.6%)	747 (31.4%)	0.99 (0.86, 1.14)	3459 (71.3%)	1392 (28.7%)	0.92 (0.83, 1.03)	3636 (73.1%)	1340(26.9%)	0.93 (0.84, 1.04)
Higher	1103 (82.7%)	231 (17.3%)	0.71 (0.57, 0.88)*	2005 (80.6%)	483 (19.4%)	0.85 (0.73, 1.00)	2135 (84.1%)	405 (15.9%)	0.70 (0.60, 0.82)*
**Religion**									
Islam	5627 (68.2%)	2624 (31.8%)	1.00	9865 (67.3%)	4789 (32.7%)	1.00	10643 (70.9%)	4366 (29.1%)	1.00
Others	675 (73.2%)	247 (26.8%)	0.75 (0.64, 0.88)*	1379 (74.7%)	467 (25.3%)	0.70 (0.62, 0.78)*	1207 (74.9%)	404 (25.1%)	0.82 (0.73, 0.93)*
**Residence**									
Rural	3753 (65.7%)	1960 (34.3%)	1.00	7039 (65.2%)	3751 (34.8%)	1.00	7555 (69.1%)	3383 (30.9%)	1.00
Urban	2549 (73.7%)	911 (26.3%)	0.91 (0.81, 1.01)	4205 (73.6%)	1505 (26.4%)	0.95 (0.88, 1.04)	4295 (75.6%)	1387 (24.4%)	0.99 (0.91, 1.08)
**Division**									
Barisal	744 (61.1%)	474 (38.9%)	1.00	1373 (70.5%)	574 (29.5%)	1.00	1294 (64.7%)	707 (35.3%)	1.00
Chittagong	1001 (62.5%)	600 (37.5%)	1.01 (0.86, 1.18)	1808 (67.8%)	859 (32.2%)	1.22 (1.07, 1.39)*	1905 (71.3%)	766 (28.7%)	0.82 (0.72, 0.94)*
Dhaka	1473 (74.6%)	501 (25.4%)	0.58 (0.49, 0.68)*	2067 (72.8%)	771 (27.2%)	0.95 (0.84, 1.09)	2218 (77.1%)	657 (22.9%)	0.62 (0.54, 0.71)*
Khulna	1023 (71.1%)	415 (28.9%)	0.67 (0.56, 0.79)*	1804 (73.3%)	657 (26.7%)	0.92 (0.80, 1.05)	1721 (71.7%)	678 (28.3%)	0.75 (0.66, 0.86)*
Rajshahi	1278 (73.2%)	469 (26.8%)	0.60 (0.51, 0.71)*	2987 (63.4%)	1727 (36.6%)	1.36 (1.21, 1.53)*	3386 (71.4%)	1359 (28.6%)	0.73 (0.65, 0.82)*
Sylhet	783 (65.5%)	412 (34.5%)	0.85 (0.72, 1.02)	1205 (64.3%)	668 (35.7%)	1.40 (1.22, 1.62)*	1326 (68.7%)	603 (31.3%)	0.82 (0.71, 0.94)*
**Decision making freedom**									
Self or joint	3053 (72.2%)	1176 (27.8%)	1.00	5475 (70.3%)	2310 (29.7%)	1.00	5717 (75.6%)	1848 (24.4%)	1.00
Others	3249 (65.7%)	1695 (34.3%)	1.23 (1.12, 1.35)*	5769 (66.2%)	2946 (33.8%)	1.14 (1.07, 1.22)*	6133 (67.7%)	2922 (32.3%)	1.40 (1.30, 1.50)*
**Media exposure**									
None	2811 (65.6%)	1474 (34.4%)	1.00	3408 (61.7%)	2116 (38.3%)	1.00	3937 (65.2%)	2104 (34.8%)	1.00
At least one	3491 (71.4%)	1397 (28.6%)	0.90 (0.82, 0.99)**	7836 (71.4%)	3140 (28.6%)	0.94 (0.87, 1.02)	7913 (74.8%)	2666 (25.2%)	0.93 (0.85, 1.02)
**Age of Respondent**			1.00 (0.99, 1.01)			1.00 (0.99, 1.01)			0.99 (0.98, 1.00)
**Age of partner**			0.99 (0.98, 1.00)			0.99 (0.99, 1.00)			1.00 (0.99, 1.00)
**Marital status**									
Before 18 years	4980 (66.9%)	2464 (33.1%)	1.00	8468 (66.1%)	4338 (33.9%)	1.00	8750 (69.4%)	3865 (30.6%)	1.00
At 18 or more	1322 (76.5%)	407 (23.5%)	0.85 (0.74, 0.97)	2776 (75.1%)	918 (24.9%)	0.87 (0.80, 0.96)*	3100 (77.4%)	905 (22.6%)	0.88 (0.80, 0.96)**

* significance at 0.5%,

** significance at 5%

The women in the richest quantile were 40% less likely to justify physical violence in any scenario compared to the poorest (Table 2). The middle and richer section also showed around 20% less chance of having such opinion. A significant opinion gap exists between the illiterate and the highly educated (graduate or more) women. Highly educated females are approximately 50% less likely to support any beating by the husband. Interestingly, primary education did not lead to difference in opinion; however, secondary education was shown to influence their opinion by a scale of 20%. Similarly, women with highly educated partners/husbands had significantly (p-value < 0.001) less chance of accepting physical violence, although the survey of 2011 did not find it to be a significant factor.

The likelihood of justifying physical spousal violence were 20-30% less for women from any other religion (Hinduism, Christianity or Buddhism) apart from Islam, compared to the women in Islamic households. Residents of Dhaka, Chittagong, Rajshahi, Khulna and Sylhet were significantly less likely to support violence towards women than the residents of Barisal, and the lowest probability is in Dhaka, the capital. Women whose decisions were made by their husbands or someone else were 1.23 times more likely to be perceived as supporting physical violence at specific circumstances compared to women who take their own decisions alone or jointly with their partners. Those who were married at the legal age (18+) are approximately 20% less likely to accept intimate physical violence in their households.

Some changes in the SESs over the years have been observed, along with women’s opinions regarding spousal physical violence in the household (Table 3). However, the surveys were limited; only three nationwide surveys were taken in between 2007 and 2014 and it is not enough to detect a trend or any trend-based modeling. Some changes are obvious; for example, the proportion of women who are illiterate and those who have only a primary education has decreased. Similarly marriages before 18 years have decreased. As the three surveys did not use the same clusters for data collection, some inconsistencies are visible. For example, in BDHS, the proportion of urban residents has decreased from 2007 to 2011 and then again from 2011 to 2014, which is not a true portrayal of overall Bangladesh [48]. However, the important consideration is the final column in Table 3, where the average proportion of women supporting violence at home is displayed. The richest group (19.8%) has the lowest acceptance rate of spousal violence compared to the other wealth groups (around 30%). Similarly, the highest educated (both women and their partner) group had the lowest average acceptance which is nearly 10-20% lower than the primary/secondary educated groups. A similar gap is observed with the decision making freedom and marital status group. Apart from these particular groups (richest, highly educated and married after 18 years), other groups had around 30% acceptance rate of household violence.

**Table 3:** Binary logistic model fitted with household SES to women’s opinion regarding physical spousal violence justification.

	Change in SES		Change in opinion (violence justified)		
SES variables	Δ(2011−2007)*	Δ(2014−2011)*	Δ(2011−2007)*	Δ(2014−2011)*	Mean over three surveys
**Wealth Index**					
Poorest	4.3%	5.3%	13.6%	-11.2%	37.8
Poor	2.7%	1.1%	3.9%	-8.0%	36.2
Middle	1.6%	5.2%	-2.3%	-14.6%	32.9
Richer	4.9%	-0.9%	-5.6%	-8.3%	30.0
Richest	-10.2%	-8.0%	-6.9%	-11.9%	19.8
**Education of respondent (women)**					
No education	-21.5%	-10.6%	14.2%	-9.8%	35.8
Primary	-1.6%	-3.0%	4.4%	-8.4%	34.3
Secondary	21.8%	5.4%	-9.0%	-8.1%	28.5
Higher	7.5%	16.3%	26.6%	-2.9%	12.7
**Education of partner**					
No education	-14.1%	-3.2%	12.6%	-10.7%	35.6
Primary	2.2%	0.4%	0.0%	-9.0%	34.3
Secondary	13.1%	1.7%	-8.6%	-6.3%	29.0
Higher	4.1%	1.3%	12.1%	-18.0%	17.5
**Religion**					
Islam	-1.2%	1.7%	2.8%	-11.0%	31.2
Others	10.9%	-13.4%	-5.6%	-0.8%	25.7
**Residence**					
Rural	5.0%	0.6%	1.5%	-11.2%	33.3
Urban	-8.2%	-1.2%	0.4%	-7.6%	25.7
**Division**					
Barisal	-11.3%	1.7%	-24.2%	19.7%	34.6
Chittagong	-7.4%	-0.6%	-14.1%	-10.9%	32.8
Dhaka	-20.0%	0.6%	7.1%	-15.8%	25.2
Khulna	-5.1%	-3.4%	-7.6%	6.0%	28.0
Rajshahi	50.5%	0.0%	36.6%	-21.9%	30.7
Sylhet	-12.3%	1.8%	3.5%	-12.3%	33.8
**Decision making freedom**					
Self or joint	2.4%	-3.6%	6.8%	-17.8%	27.3
Others	-2.0%	3.2%	-1.5%	-4.4%	33.5
**Media exposure**					
None	-28.3%	8.4%	11.3%	-9.1%	35.8
At least one	24.8%	-4.2%	0.0%	-11.9%	27.5
**Marital age**					
Before 18 years	-4.4%	-2.2%	2.4%	-9.7%	32.5
At 18 or more	19.1%	7.6%	6.0%	-9.2%	23.7

* $\begin{array}{c} \Delta =\frac{Y_{n}-Y_{\left(n-1\right)}}{Y_{\left(n-1\right)}}\times 100 \end{array}$

## Discussion

Physical spousal violence, a part of IPV, is often considered to be a ‘right’ of the husband or partner to ‘correct’ his wife in Asia, particularly in poor illiterate households [49]. Household economic status and women’s participation in earning determine whether she will remain a subordinate dependent part of the family or her opinion will be valued [50]. It is interlinked with her education and marital age, which determine her maturity [51, 52]. Unfortunately, due to the high prevalence of child marriage and poverty, women are commonly beaten in patriarchal households by their husbands in Bangladesh [33, 53]. This study reached the same conclusion that the most vulnerable households are characterized by low income, illiteracy and child marriage, where women tend to accept their fate of being beaten by their husband/partner.

We found that acceptance of physical spousal violence is more likely among women belonging to households following Islam as well as those who marry at a young age. The culture of Bangladesh is traditionally conservative and Islamic views in that context do not encourage women to speak against the ‘expected norm’ that their mothers or grandmothers have followed, which incidentally leads to acceptance of spousal physical violence [24, 54, 55]. The situation is worsened with high prevalence of dowry that forces women to marry early, so they are treated as children in their in-laws’ house, which narrows their views on empowerment or resisting any spousal violence [56–58]. Naved and Persson, 2010 [59] showed that absence of dowry lowered the likelihood of beating wives compared to marriages where dowry was demanded and fully paid in Bangladesh. Thus, in traditional patriarchal society, where women are forced to marry early, the victims are likely to accept or at least agree on physical violence in least agree on certain cases.

Women’s freedom of decision is also entwined with their status in the household. If a woman is educated, employed, and married to an educated husband, then she is more likely to make major household decisions alone or jointly with her husband [60, 61]. However, the traditional mind set of the patriarchy excludes women in important decisions and more often her life purposes are settled by the husband and/or mother-in-law [62, 63]. Thus, women’s liberation from the binding cultural stereotypic norms could be an intervention strategy [49]. We found spatial variation in Bangladesh, where currently the Barisal division showed highest vulnerability, followed by Sylhet. These division wise differences are influenced by the same SESs, the gap in education and inequality; for example, the Sylhet division lags behind in education and Barisal in economy [64–66].

In this study, we did not find any significant difference in opinion between women residing in urban and rural areas of Bangladesh. However, spousal physical violence is more common in rural than urban areas [67, 68]. One explanation could be that a higher number of slums in urban areas share same mentality; however, they are less victimized [33]. Lack of education and wealth force women to assume a dependent life and remain silent regarding IPV, which they gradually accept over time [69]. It is also important to note that the significant variables (and the magnitude of their scales) were consistent from 2007 to 2014, which shows that the vulnerable households display the same characteristics. A number of intervention studies are required to formulate a policy that targets the most vulnerable households in Bangladesh, particularly those where women themselves finds justification for spousal physical violence.

The study has come to the conclusion that the spousal violence scenario has seen little change in Bangladesh over the past few years (2007 onwards), with a prevalence rate from 31.3% in 2007 to 28.7% in 2014. The most important finding is that the household SESs did not change much in these surveys, with households in the vulnerable groups showing that around 30% accept spousal violence. The poor households with illiterate inhabitants are the most vulnerable with a raised level of spousal physical violence. These should aid policymakers to ascertain an intervention focusing on those households. Success of the interventions can be assessed based on analyses like this study, where we could expect a shift in the magnitude of spousal physical violence. The intervention would be expected to be conducted by both the government and the non-governmental organizations (NGO), who are advocating women’s rights in Bangladesh [70]. As our data allowed only a characterization of the most vulnerable households, we cannot speculate on future interventions. As mentioned above, the focus in this paper has been solely on women; however, an intervention scheme should be devised to target both men and women. Several experimental studies are required to find a working intervention model for Bangladesh to address spousal domestic violence.

This study is limited by the lack of qualitative analysis. A number of focus group discussions in the areas where the quantitative data were taken could have substantiated the interpretation and discussion. A district wise national data set with opinion from both men and women of Bangladesh would further specify those target areas that would benefit from attention by policymakers. Furthermore, other household SES, for example the number of household members, total children ever born, NGO membership could be considered in future models. Caution must be taken while interpreting both ‘Decision making freedom’ and ‘Media exposure’ variables, defined by the authors, based on a series of questions, which are not an official scale of BDHS.

## Conclusions

This study analyzed the opinion of Bangladeshi women regarding their justification behind spousal physical violence and the contribution of household SES in that judgment. The three most recent surveys were analyzed to characterize the most vulnerable households in Bangladesh, where women tend to accept violence from their partner. The poorest households where both husband and wife are illiterate and the brides were married at a young age (before 18 years) are the most vulnerable. Moreover, the freedom of decision making is low for such women in the patriarchal society of Bangladesh.

The traditional patriarchal society of Bangladesh generally considers women as subordinate to men, where their responsibility lies with their fathers and later on their husbands. They are, in most cases, taught to accept spousal physical violence as part of their daily life, as this has been ongoing for generations. However, the status quo is improving in Bangladesh, especially in urban areas, with the spread of education and exposure to wider worlds. Nevertheless, a high quantity of households (approximately 30%) remain, where women themselves find justification for such violence. To compile a policy of intervention, the most vulnerable households must be characterized and identified, particularly the geographical areas where women experience the spousal violence.
